# Towards efficient cancer immunotherapy: advances in developing artificial antigen-presenting cells

**DOI:** 10.1016/j.tibtech.2014.06.007

**Published:** 2014-09

**Authors:** Loek J. Eggermont, Leonie E. Paulis, Jurjen Tel, Carl G. Figdor

**Affiliations:** Department of Tumor Immunology, Radboud University Medical Centre and Radboud Institute for Molecular Life Sciences, Nijmegen, The Netherlands

**Keywords:** artificial antigen-presenting cell, synthetic dendritic cell, immunotherapy, cancer

## Abstract

Active anti-cancer immune responses depend on efficient presentation of tumor antigens and co-stimulatory signals by antigen-presenting cells (APCs). Therapy with autologous natural APCs is costly and time-consuming and results in variable outcomes in clinical trials. Therefore, development of artificial APCs (aAPCs) has attracted significant interest as an alternative. We discuss the characteristics of various types of acellular aAPCs, and their clinical potential in cancer immunotherapy. The size, shape, and ligand mobility of aAPCs and their presentation of different immunological signals can all have significant effects on cytotoxic T cell activation. Novel optimized aAPCs, combining carefully tuned properties, may lead to efficient immunomodulation and improved clinical responses in cancer immunotherapy.

## Highlights

• Active immunotherapy is promising for the development of potent cancer therapeutics.
• Various types of artificial antigen-presenting cells (aAPCs) may be used as ‘off-the-shelf’ products to induce antigen-specific T cell activation both *ex vivo* and *in vivo.*
• Size, shape, cytokine delivery mechanism, ligand composition, ligand mobility, and ligand positioning on aAPCs all have significant effects on T cell activation, and therefore should be taken into account when designing novel constructs.

## Advances in cancer immunotherapy

**Figure 1:**
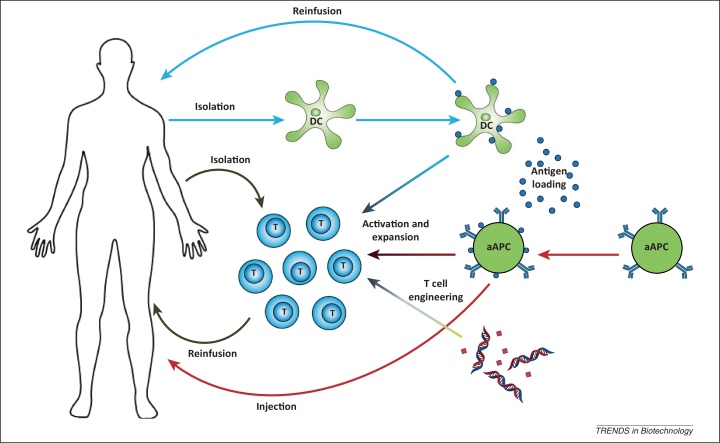
Different strategies for active cancer immunotherapy. T cell activation can be induced either *ex vivo* or *in vivo* by autologous dendritic cells (DCs; blue arrows) or artificial antigen-presenting cells (aAPCs; red arrows), or by engineering of T cells through transgenic delivery of T cell receptors (TCRs; green arrow) and lifetime engineering, for example using small-molecule inhibitors (red diamonds). *Ex vivo*-activated autologous T cells can be adoptively transferred into patients (grey arrows) to specifically kill cancer cells. Alternatively, injection of APCs can lead to *in vivo* aAPC immunotherapy without the need for autologous cell cultures (red arrows).

In cancer immunotherapy, the immune system is either passively or actively exploited to target and kill cancer cells. In this way, higher specificity for malignant cells may be achieved than with conventional cancer therapeutics. This approach thus avoids off-target toxicities while still inducing highly potent anti-cancer responses. By targeting tumor cells or their microenvironment, passive immunotherapy exploiting monoclonal antibodies has proven beneficial clinical effects in several malignancies 1, 2. In active immunotherapy, immune cells are stimulated and instructed to actively fight cancer and although more challenging, this approach is extremely promising. Active immunotherapy is highly dependent on efficient stimulation of antigen-specific immune cells, such as killer T cells and antibody-producing B cells. In adoptive T cell transfer, isolated autologous tumor-specific T cells are expanded *ex vivo* and, after sufficient stimulation, are reinfused into the cancer patient, where these cells are expected to elicit potent anti-tumor responses (Figure 1, gray arrows) [3].

A more recent trend in adoptive T cell transfer exploits molecular biology approaches to create more active T cells with higher target affinities and prolonged lifetimes (Figure 1, green arrow). For this purpose, T cells have been constructed that express either transgenic T cell receptors (TCRs) with increased affinity for their peptide–major histocompatibility complexes (MHC) complexes, or chimeric antigen receptors that can target antigens independent of MHC through antibody-derived ligand-binding domains. Engineered T cells have been clinically applied and show good results, but several issues need to be addressed, including on-target and off-target toxicities, undesirable immune responses to chimeric antigen receptors and engineered TCRs, and the possibility of transformation, either because of virus-related insertional mutagenesis or misguided T cell lifetime engineering. Furthermore, engineered T cells do not differentiate into memory cells and therefore no immunological memory is created. These fundamental issues need to be resolved before engineered T cells can be broadly implemented as anti-cancer therapy 4, 5.

*In vivo*, induction of T cell responses is highly dependent on interactions with professional antigen-presenting cells (APCs), in particular dendritic cells (DCs), which present tumor-specific antigens. Therefore, to induce *in vivo* T cell activation, cancer patients are vaccinated with APCs [6]. In contrast to engineered T cells, these approaches use physiological interactions, which minimizes the risk of serious adverse side effects. Natural APCs, in particular DCs, are well equipped to induce efficient activation and expansion of tumor antigen-specific naïve T cells, which can lead to induction of large populations of T cells, including CD8^+^ cytotoxic T lymphocytes (CTLs) that can kill cancer cells antigen-specifically (Figure 1, blue arrows). Several studies now indicate that the use of natural APCs in cancer treatment is associated with a beneficial clinical outcome with minor adverse side effects, emphasizing the promise of active immunotherapy 3, 6, 7, 8.

Unfortunately, the use of natural APCs such as DCs over the years has also uncovered several serious limitations. Lack of knowledge of the optimal antigen-loaded DC combined with deleterious effects of immunosuppressive factors in the tumor microenvironment may be responsible for the mixed results observed in clinical trials 9, 10, 11, 12. In addition, isolation and *ex vivo* stimulation of autologous DCs proved time-consuming and expensive, and the quality of *ex vivo*-generated DCs can be variable 9, 13, 14. The use of patient-derived autologous DCs therefore limits standardization of DC-based treatment protocols 9, 14.

## Artificial APCs for T cell activation

To overcome the disadvantages and difficulties in use of autologous APCs, artificial APCs (aAPCs) have been developed as an alternative for both *ex vivo* and *in vivo* induction of tumor-specific CTLs (Figure 1, red arrows) 15, 16. Whereas natural APCs may be influenced by the tumor microenvironment and unknown signaling moieties may be present on their surface, artificial presentation of antigens allows for better defined systems with more control over the signals presented. Furthermore, the use of aAPCs does not require time-consuming and expensive cell-culture strategies and can be developed into an off-the-shelf technology 14, 15. However, aAPCs are not equipped with a machinery to actively migrate into tissues. In this review, advances in aAPC development are discussed for both *ex vivo* and *in vivo* application in cancer immunotherapy.

## Cell-based aAPCs

Genetically modified xenogeneic or allogeneic cells, such as *Drosophila* cells, murine fibroblasts, and human erythroleukemia cells, have been used as aAPCs 17, 18, 19. These cells are easier to handle and are better defined than DCs, allowing for more control over the signals delivered. In addition, cellular aAPCs are stable cell lines that can be stored for extended times and can thus be obtained from a readily accessible source [16]. However, a major disadvantage is their allogeneic nature. The use of human cell-based aAPCs has recently been extensively reviewed [20], so this review is restricted to the use of acellular aAPCs for active cancer immunotherapy.

## Acellular synthetic aAPCs for efficient expansion of CTLs

**Figure 2:**
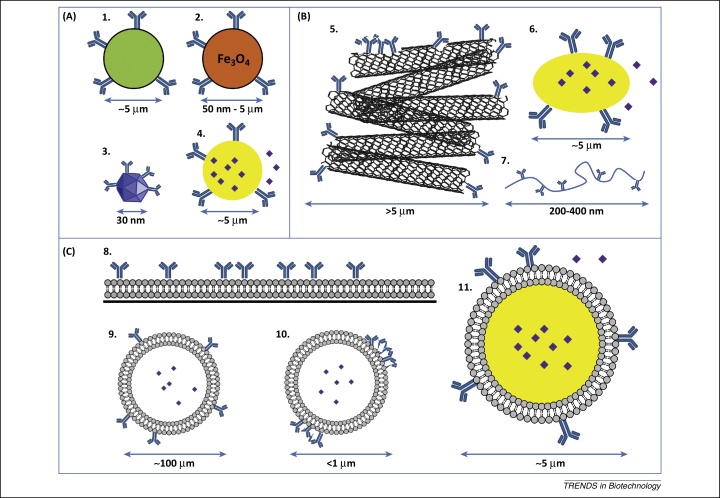
Different types of synthetic artificial antigen-presenting cells (aAPCs). **(A)** Rigid spherical particles: 1, polystyrene latex microbeads; 2, magnetic nano- and microparticles; 3, nanosized quantum dots; and 4, poly(lactic-co-glycolic acid) (PLGA) microspheres. **(B)** Nonspherical particles: 5, carbon nanotube bundles; 6, ellipsoid PLGA microparticles; and 7, nanoworms. **(C)** Fluidic lipid bilayer-containing systems: 8, 2D-supported lipid bilayers (2D-SLBs); 9, liposomes; 10, RAFTsomes/microdomain liposomes; and 11, SLB particles.

Although cellular aAPCs can induce high expansion rates of CD8^+^ T cells, they do not easily allow for specific control of expression levels of T cell-activating signals. In addition, non-tumor antigens and other stimulatory or inhibitory molecules may be present on cellular aAPCs 13, 21. To better define the delivery of distinct signals and circumvent the use of allogeneic cells, acellular aAPCs have been developed. Compared to cellular aAPCs, acellular aAPCs allow for more stringent control over the signals delivered and are attractive tools because of their relatively easy preparation. These synthetic entities can be designed to be either nonspecific or antigen-specific by presenting T cell-activating antibodies (such as anti-CD3) or peptide–MHC complexes, respectively. In general, aAPC approaches have focused on induction of CD8^+^ CTLs through MHC I stimulation, because these cells are capable of antigen-specific tumor cell lysis. Other immune cells, such as CD4^+^ T helper cells, can assist in shaping the anti-cancer immune response by helping in the activation of CTLs. Therefore, efforts have also been made, albeit to a lesser extent, to activate these cells via MHC II. Artificial APCs comprising various sizes, surface ligand distributions, ligand mobilities, and shapes have been developed, and these properties can all affect T cell activation. The wide variety of acellular aAPC structures (Figure 2, Table S1 in the supplementary material online) reflects different attempts to mimic different aspects of natural DCs.

## Rigid spherical microsized aAPCs

To mimic natural APCs, several cell-sized, rigid, bead-based aAPCs have been developed. Because of their homogenous size distribution and straightforward functionalization, these beads have been extremely useful in determining the various signals necessary for T cell activation. In addition, these more simplistic systems have been used to induce T cell activation for clinical purposes [16].

### Latex microbeads

To induce T cell expansion, spherical polystyrene beads can be coated with antibodies against CD3 and CD28. It was shown that T cell activation was optimally induced by microbeads ranging in size between 4 and 5 μm [22]. Ligand density and bead size, rather than the amount of beads, were important parameters for T cell activation. However, these nonspecific particles could only induce long-term proliferation of CD4^+^ helper T cells, and did not support the growth of CD8^+^ CTLs for extended culture periods, indicating that CD8^+^ T cells require additional stimulation to maintain their effector functions [23]. Furthermore, anti-CD3 stimulation has been associated with a loss of antigen specificity when expanding enriched CD8^+^ T cell populations [19]. These reported problems can be avoided by replacing anti-CD3 with specific peptide–MHC complexes in combination with co-stimulatory signals [24].

These beads were successfully used to produce large numbers of functional antigen-specific CTLs against different targets, including melanoma antigens TRP-2 and Mart-1 16, 25. To induce long-term expansion of CD8^+^ T cells using latex microbeads, cells require the presence of IL-2. It was recently demonstrated that IL-2 may also be replaced by IL-21 or a combination of IL-15 and IL-21, which leads to unique functional CTL phenotypes 26, 27. Improved stimulation of T cells can also be achieved by coating beads with anti-4-1BB or 4-1BBL, ICAM-1, and CD83 25, 28, 29.

A single peptide–MHC complex has low intrinsic affinity for its specific TCR [30]. To enhance this affinity, multimers of MHCs have been developed, such as human leukocyte antigen (HLA) tetramers 31, 32, 33. In addition, IgG–HLA dimers, which consist of IgG molecules containing two MHCs that can be easily uploaded with any desired antigen, have been developed. In this way, aAPCs can be made antigen-specific by uploading IgG–HLA dimers with any desired epitope [34]. Dimers have also been applied to synthetic nanoparticles, including dextran-coated iron oxide magnetic particles (50–100 nm) and dextran-coated quantum dots (30 nm). Although previous research using bead-based systems indicated only very low T cell activation below 3 μm [22], these nano-aAPCs showed improved *in vivo* efficacy compared to microsized beads. Although first studies demonstrated that these particles exhibited similar or even improved *ex vivo* T cell activation, the same group later showed better activation for micro-aAPCs using this type of particle [35], which is in accordance with previous literature [22]. Their ability to stimulate T cells despite their small size may be attributed to the use of IgG–MHC, which contains a flexible hinge region [36]. In general, it is thought that microbeads are better suited to stimulate T cells because, owing to the lower curvature of the surface, a microbead can make more interactions with the cell than a nanosized spherical bead.

### Polystyrene-coated magnetic microbeads

Because polystyrene particles are not biodegradable and may be toxic or induce embolisms *in vivo*, they must be removed from the CTL population before *ex vivo*-expanded T cells are infused into patients [9]. For this purpose, microsized magnetic latex-coated beads have been developed by coating an iron oxide core with polystyrene, which allows for straightforward removal of aAPCs by magnetic depletion before reinfusion of CTLs. These beads were initially coated with anti-CD3 and anti-CD28 for nonspecific CD4^+^ T cell amplification [37]. Again, replacement of anti-CD3 with peptide–MHC complexes results in antigen-specific T cell expansion [31]. Similar to nonmagnetic latex beads, these particles can be easily prepared and are readily available [9].

Importantly, T cells expanded *ex vivo* using anti-CD3/anti-CD28-coated magnetic beads have been applied in Phase I clinical trials in patients with metastatic breast cancer, chronic myeloid leukemia, and carcinomas. Although infusion of expanded CTLs resulted in mixed anti-tumor responses, in some cases bead-expanded cells induced tumor regression or even complete remission, indicating that adoptive T cell transfer using bead-based aAPC-expanded T cells could be an effective cancer treatment. However, these trials also showed a risk of developing non-tumor-specific cytotoxicity or graft versus host disease 38, 39, 40, 41.

### Biodegradable poly(lactic-co-glycolic acid) microparticles

Bead-based aAPCs can provide strong proliferative signals to CD8^+^ T cells. Natural APCs provide a third signal (Box 1, Box 2) besides MHC-mediated antigen presentation and membrane receptor-based co-stimulation, in the form of soluble secreted cytokines. CD8^+^ T cells require continuous paracrine delivery of cytokines such as IL-2 during the first few hours for proper activation, and these are initially provided by APCs and later by CD4^+^ helper cells. Besides paracrine delivery, trans-presentation of IL-2 on CD25 also plays an important role in initial activation of naïve T cells [42]. At later time points, CTLs also require cytokine stimulation to maintain proliferation, but high IL-2 concentrations and exposure for extended times may negatively impact T cells [43]. When using polystyrene beads, cytokines have to be added to the culture, leading to high overall concentrations and possible side effects due to co-injection of IL-2 during reinfusion of T cells into patients [9]. Implementation of mechanisms that allow for paracrine cytokine release by aAPCs may therefore further improve their potential to activate and differentiate T cells. In particular, cytokine release may be essential for the development of functional CD8^+^ T cells that can generate potent immune responses. This may be the one most important characteristics of an aAPC.

Box 1 **Signals for T cell activation**

**Activation signal 1: antigen recognition**

T cell activation occurs after a T cell receptor (TCR) recognizes a specific peptide antigen presented on MHC complexes of an antigen-presenting cell. In general, extracellular peptides are presented on MHC class II, which is recognized by the TCR in conjunction with the CD4 T cell co-receptor, whereas MHC class I carries peptides derived from intracellular proteins and are recognized by the TCR in conjunction with a CD8 T cell co-receptor. Ligation of the TCR by a peptide–MHC complex or binding of agonistic antibodies directed towards CD3 leads to activation of the CD3 signal-transduction complex, which transduces essential signals necessary for activation of the T cell.

**Activation signal 2: co-stimulation**

To become fully activated, T cells require a second signal next to TCR-mediated antigen recognition. This antigen-independent signal is provided in the form of co-stimulatory molecules that are upregulated on antigen-presenting cells when they encounter stress, infection, or cellular damage. These molecules can interact with receptors on the T cell, of which CD28 is the best studied. This receptor interacts with co-stimulatory molecules B7-1 (CD80) or B7-2 (CD86) on APCs, which can also transduce inhibitory signals by ligation of CTLA4 on the T cell. Other co-stimulatory receptors on T cells include inducible co-stimulator (ICOS), CD27, and 4-1BB (CD137), which bind to ICOS-L, CD70, and 4-1BBL, respectively.

**Activation signal 3: cytokines**

T cell activation and differentiation can be further directed through binding of cytokines to cytokine receptors. These cytokines can be produced by either APCs or CD4^+^ T helper cells and, especially for CD8^+^ T cells, are essential for cell survival and productive immune responses. IL-2, the most important cytokine for CD8^+^ T cell survival, is secreted in low amounts by APCs during initial T cell encounter, and is produced in larger amounts by activated CD4^+^ T cells. Other cytokines that can assist in T cell activation include IL-12, IL-15, IL-21, and type I interferons (IFNα/β). In particular, IL-12 and IFNα/β seem to be essential for effective T cell function. In addition to immunostimulatory cytokines, immunoinhibitory cytokines such as IL-4, IL-5, and IL-10 are capable of dampening the immune response or can lead to tolerance. Therefore, signal 3 is regarded as the most important in shaping the immune response.

Box 2 **Signal transmission by APCs**

For efficient induction of tumor-specific CTLs, several signals need to be transferred from APCs to naïve T cells (Figure I). Antigen recognition represents the first signal, which occurs through the interaction of specific T cell receptors (TCRs) with peptide–MHC complexes on APCs. APCs can contain two different types of MHCs. MHC class I molecules present antigens derived from intracellular proteins and bind to T cells expressing the CD8 co-receptor, whereas MHC class II molecules, which present extracellular peptides, can bind to CD4^+^ T cells. Activated CD8^+^ T cells, or CTLs, are capable of antigen-specific cell lysis, whereas CD4^+^ T cells, known as T helper cells, release cytokines to stimulate CTL activation and antibody production by B cells. In artificial systems, peptide–MHC complexes can be replaced by antibodies binding the CD3 subunit of the MHC complex, which leads to non-antigen-specific T cell activation. Besides signal 1, a second signal, co-stimulation by APC cell-surface molecules, is passed on to naïve T cells. This co-stimulation is necessary for proper T cell activation. CD80 and CD86, which bind to CD28 on T cells, are the most prominent co-stimulatory signals. Additional co-stimulatory molecules, such as 4-1BB and CD83, are also expressed on APCs. Finally, to induce more efficient expansion and specific differentiation of T cells, immune cells release cytokines, such as IL-2, IL-15, and IL-21, which can be viewed as the third signal. In addition, T cell–APC interactions are guided by adhesion molecules. Important adhesion molecule interactions include those of ICAM-1 (CD54) and LFA-3 (CD58) on APCs binding to LFA-1 and LFA-2 on T cells, respectively. Similar to natural APCs, the success of aAPCs depends to a great extent on efficient presentation of these different signals to naïve T cells 6, 9, 15.

**Figure I:**
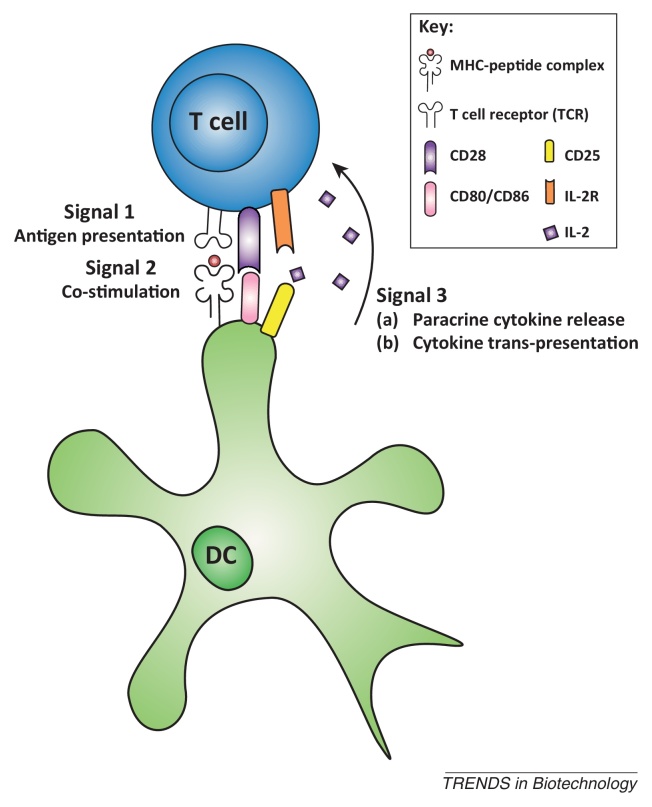
Different signals leading to induction of T cell activation and expansion.

To facilitate release of cytokines or other soluble factors from aAPCs, biodegradable systems have been developed. These biodegradable aAPCs present signals on their surface, similar to polystyrene particles, but combined with slow release of IL-2 or other soluble molecules of interest. Particles composed of the biodegradable co-polymer poly(lactic-co-glycolic acid) (PLGA) have been extensively applied in slow release systems. Although it can be challenging to stably present molecules on the surface of biodegradable PLGA particles owing to loss of surface-bound molecules as the particle degrades [15], incorporation of avidin–palmitate conjugates facilitated incorporation of all three signals in one aAPC 44, 45. Surface ligand presentation of these IL-2-releasing particles was stable for 20 days, and they led to significantly higher induction of IFNγ secretion by murine and human T cells compared to magnetic beads in the presence of soluble IL-2 and reached peak activation profiles at lower aAPC concentrations [45]. Furthermore, it was shown that this paracrine release by aAPCs, in contrast to exogenous addition, can induce local accumulation of IL-2 near the contact area with the T cell, thereby significantly altering the activation and proliferation of CD4^+^ CD8^+^ T cells, leading to apoptosis of CD4^+^ T cells and enhanced proliferation of CD8^+^ killer T cells 43, 46. It is likely that the high synaptic concentration that results from paracrine IL-2 delivery can be detected by the low-affinity IL-2 receptor, which is constitutively expressed by T cells. After 24 h of antigen stimulation, T cells can also express the high-affinity IL-2 receptor, and lower amounts of IL-2 may be needed [43]. Similar to latex beads, PLGA particles of 6–10 μm in size were most effective inducers of T cell activation and expansion *ex vivo*[45].

Cytokines are essential for induction of potent CD8^+^ T cells (Box 3), but it is difficult to design non-biodegradable aAPCs, such as latex microbeads, capable of releasing cytokines for extended periods of time. To circumvent this problem, cytokines may be presented by surface receptors similar to CD25, as also occurs on DCs [42]. However, it is questionable whether sufficiently high concentrations of cytokines will be available. Alternatively, agonistic antibodies may be used, which could also be bound to non-biodegradable aAPC structures. So far, only biodegradable aAPCs have been used for cytokine delivery, which may induce a more natural response via paracrine delivery. However, it should be noted that because of their biodegradable nature, these aAPCs might release their stimulatory surface molecules, which may influence their efficacy for T cell activation.

Box 3 **Cytokine delivery for T cell activation**

Inflammatory cytokines are essential for the survival (in particular IL-2), proliferation, and functional differentiation of CD8^+^ T cells. However, most aAPCs are not capable of releasing sufficient cytokine levels and require additional cytokines in cell culture, which may not be as effective as either paracrine delivery or trans-presentation (by CD25) by APCs [42]. This underscores the importance of incorporating cytokine release systems in aAPC designs. IL-2 may also induce regulatory T cells that inhibit immune responses, so additional cytokines are required to further induce effective T cell responses and avoid tolerance (non-responsive T cells). Therefore, development of aAPCs that deliver other soluble factors, or can trigger cytokine receptors in different ways (e.g., by using antibodies), could lead to major improvements in T cell survival and function. Interesting candidates include IL-12 and type I IFN, usually produced by DCs, which are responsible for prolonged T cell survival. These cytokines are necessary for memory T cell development and strong effector function of CD8^+^ T cells. Another interesting combination of cytokines is IL-15 and IL-21. IL-15 is similar to IL-2 but does not lead to CD8^+^ T cell apoptosis. These cytokines have been used to rescue tumor-reactive CD8^+^ T cells *in vivo* [87]. In addition, it was demonstrated that IL-21 acts in synergy with IL-15 [27]. Additional cytokines, such as IFNγ and IL-7, can be secreted by the T cell itself or by stromal cells in lymph nodes and may therefore not be essential for aAPC-mediated delivery.

## Importance of ligand mobility and pre-clustering for T cell activation

Although rigid spherical beads can be efficiently used for T cell stimulation, the membrane of natural APCs is much more dynamic than the outer surface of latex-coated and PLGA particles. To more closely mimic natural systems, lipid bilayer surfaces and liposomes have been used as aAPCs, thereby demonstrating a significant effect of membrane fluidity and receptor pre-clustering on T cell activation.

### 2D surface membrane mimics

The immunological synapse (IS) was originally identified as a membrane structure of approximately 70 μm^2^ that forms upon APC–T cell contact and consists of peptide–MHC complexes interacting with TCRs, surrounded by a ring of interacting adhesion molecules. More recent research indicates that this is a dynamic structure formed after TCRs cluster together in microclusters that eventually move towards the IS centre. It was recently shown that TCR-containing vesicles excreted within the IS are also important for signal transmission [47]. In general, efficient CD4^+^ and CD8^+^ T cell activation is associated with the formation of a functional IS [48]. Synthetic 2D cell-surface mimics have played an important role in elucidation of the structure, function, and mechanisms of IS formation [48]. Planar membrane surfaces can easily be functionalized with various ligand compositions and allow for the use of high-resolution microscopy techniques, making it an attractive tool to study cell membrane interactions [49].

To examine the effect of membrane fluidity on synapse formation and T cell activation, lipid bilayers can be deposited on glass surfaces to form supported lipid bilayers (SLBs). Variation of the membrane fluidity of these lipid bilayers revealed that more fluid membranes are better capable of forming ISs, leading to improved CD4^+^ T cell activation compared to more rigid membranes [50]. In addition, 2D surface patterning has been used to study the effect of spatial organization of anti-CD3 and anti-CD28 on CD4^+^ T cell activation. Lithographic definition of the positions of proteins on a surface revealed that spatial organization is important for efficient IS formation and thus for T cell activation [51]. T cell responses were most efficient when co-stimulating molecules (anti-CD28) surrounded anti-CD3 compared to other organizational arrangements 51, 52. Incorporation of protein patterns onto aAPCs may therefore provide a new level of control over T cell proliferation [53]. Although there may be small differences between ISs of CD4^+^ and CD8^+^ T cells, the general IS organization and dynamics are similar between the various T cell subsets [54].

Although synthetic 2D cell surfaces, and in particular 2D-supported lipid bilayers (2D-SLBs), were useful in clarifying the mechanisms of T cell activation, at present they are less suitable as ‘off-the-shelf’ aAPCs owing to their fragility and limited lifetime [53]. Furthermore, it should be noted that most cell-surface membrane mimics used to date lack organizers, such as a cytoskeleton, which play an important role in the distribution of transmembrane molecules, usually resulting in a non-random distribution but organization into microdomains, whereby groups of surface receptors are clustered together.

### Liposomes, RAFTsomes, and microdomain liposomes

To facilitate T cell activation in a more natural context similar to the fluid membrane interactions of natural APCs and T cells, MHC-containing liposomes have been generated. Phospholipid bilayer vesicles with randomly distributed peptide–MHC complexes have been used to study the physiological mechanisms of CD4^+^ T cell activation 55, 56, but have not been extensively used as aAPCs 16, 55.

It has been shown that pre-clustering of peptide–MHC complexes in lipid raft microdomains on APCs dramatically increases their antigen presentation efficacy [57]. It is likely that this allows for better docking to their counterpart TCRs on T cells, which are initially also distributed as microdomains. Therefore, liposomes containing lipid raft microdomains with pre-clustered MHC complexes have been designed, which aids in IS formation. These liposomes, also known as RAFTsomes, can be made by incorporating DC-derived lipid rafts in liposomal phospholipid bilayers [58]. However, at present these RAFTsomes are not as efficient as natural DCs or other aAPCs in stimulating CD4^+^ T cell proliferation [58], probably because they lack cytoskeleton-mediated surface organization. Another method for pre-clustering molecules on liposomes makes use of ganglioside GM1-containing liposomes. A high density of ligands can be created by taking advantage of the high affinity of the cholera toxin B subunit for GM1, which is a component of lipid rafts. These microdomain aAPCs have been used for *ex vivo* T cell activation studies, and showed improved antigen-specific CD4^+^ T cell stimulation compared to liposomes with randomly distributed MHC molecules [59]. Importantly, it has been shown that these aAPCs are better equipped to stimulate CD8^+^ T cells compared to magnetic beads, leading to higher numbers and more efficient CTLs specific for MART-1 in melanoma skin cancer [60].

### 3D-supported lipid bilayers (3D-SLBs)

Although the fluidity of liposomes is a great advantage for T cell stimulation, they are substantially less stable than solid particles. This problem may be solved by using solid particles as a scaffold for a lipid bilayer. These SLB particles combine the fluidity of liposomal bilayers with advantageous properties of solid particles, such as their high stability [15]. Several 3D-SLBs have been constructed, including lipid bilayers on a core of hydrogel, PLGA, or silica 61, 62, 63. Using cell-sized silica beads coated with a lipid bilayer, it was shown that 3D-SLBs can boost CTL responses in antigen-primed T cells more efficiently than liposomes. By contrast, 3D-SLBs were not able to initiate primary T cell expansion, probably due to lack of soluble factors such as IL-2 [64]. In another approach, lipid bilayers were isolated from Ag-bearing cells and adsorbed onto latex microspheres. Perhaps cytoskeleton-organized microdomains might be grafted onto these latex spheres, but no significant change in T cell activation was observed when compared to rigid latex beads [22].Alternatively, tumor-cell derived plasma membrane vesicles have been deposited on both silica and latex microbeads, leading to large multivalent immunogens, which have an increased immunostimulating activity compared to the nanosized tumor-derived vesicles [65]. Although the use of 3D-SLBs is a promising approach, so far no recent reports have been made on SLBs that do not need membranes extracted from tumor cells for T cell activation.

## Importance of aAPC shape

Most aAPC systems use spherical particles to stimulate T cells. However, natural APCs, especially DCs, are not spherical, and therefore the aAPC shape may be modified to increase the contact area with T cells. It will be interesting to design and test differently shaped particles and examine nanoclustering of ligands. This may assist in more efficient TCR nanoclusters on the T cell surface. As described below, shape appears to be an important parameter for T cell activation that should be taken into account when designing aAPCs.

### Ellipsoid PLGA microparticles

Besides spherical forms, PLGA-based microparticles have also been used as non-spherical particles [45].To closely mimic the natural situation and increase the particle contact area, ellipsoid PLGA-based aAPCs were prepared using film-stretching methods 66, 67. Interestingly, particle shape had a significant effect on stimulation of T cells by aAPCs. Elongated particles with a more ellipsoid shape were more efficient as aAPCs than spherical particles and induced stronger CD8^+^ T cell proliferation. These findings may be explained by the fact that T cells have more and larger biomimetic interactions with these aAPCs, thereby favoring the flatter, longer side of the ellipse, which may provide a larger IS-like contact area [67].

### Carbon nanotube bundles

To both increase the surface area of particles and facilitate more options for surface modifications, single-walled carbon nanotubes have been used for efficient T cell stimulation [68]. Using anti-CD3-coated tubes, large surface particles evoked higher aspecific T cell activation and IL-2 production than polystyrene beads. These functionalized nanotubes appear to cluster into large microsized aggregates with a high surface area, perhaps mimicking cell-surface microdomains [68]. As already demonstrated for fluid membranes and microdomain liposomes, nanopatterning is an extremely important issue that deserves further study to improve aAPC-mediated T cell activation. Chemical modification of nanotubes might also lead to local clustering of antibodies on their surfaces, with a positive effect on T cell activation. Furthermore, this modification causes the nanotubes to have a negative surface charge resembling that of natural APCs, which might also help in interactions with T cells [69]. It was recently demonstrated that peptide–MHC complexes can also be stably linked to nanotubes, which makes it possible to use these aAPCs for antigen-specific T cell stimulation [70]. By combining a large surface area for interaction, pre-clustered antibodies, and a negative surface charge, these carbon nanotube bundles seem extremely potent for *ex vivo* T cell activation [68].

### Nanoworms

Lipid bilayer aAPCs have shown that membrane fluidity, which allows ligand motility, positively affects T cell activation. In addition, the particle shape appears to have a great influence on this process, resulting in enhanced responses when T cells have a larger contact area and can thus better fit onto the aAPCs. A novel promising approach incorporates both of these features into an aAPC system exploiting so-called nanoworms, composed of semi-flexible filamentous polymers comprising poly(isocyano dipeptide) with oligo(ethylene oxide) side chains, which can be decorated with molecules for antigen presentation and co-stimulation in a highly controlled fashion [71]. Anti-CD3 nanoworms composed of 200–400-nm-long polymers induced more efficient and more sustained T cell responses compared to anti-CD3 PLGA microparticles. This can probably be attributed to the semi-flexible nature of these polymers, which may assist in the formation of TCR nanoclusters on the T cell surface. Attachment of peptide–MHC complexes and various co-stimulatory molecules to a polymer backbone could lead to an aAPC that is highly promising for induction of both *ex vivo* and *in vivo* T cell responses [71].

## aAPC development for *in vivo* immunotherapy

*Ex vivo* expansion and subsequent injection of autologous CTLs is one approach for induction of anti-tumor immune responses. However, the survival and function of these cells can be highly variable after reinfusion into patients 33, 72, 73. In addition, culturing of autologous T cells is a time-consuming, labor-intensive, and costly procedure [74]. Alternatively, to avoid the use of autologous cells, aAPCs can be administered directly into patients to stimulate CD8^+^ T cell responses *in situ*, allowing for true ‘off-the shelf’ cancer immunotherapy.

When considering *in vivo* T cell targeting through injection of aAPCs, several additional properties besides high T cell stimulation should be taken into account, including the pharmacokinetics and biocompatibiliy of the system. Therefore, the optimal size, surface modifications, shape, and targeting moieties have been extensively investigated for biomaterials used in drug delivery 67, 75, 76, 77, 78.

Several aAPCs have been tested for *in vivo* induction of tumor cell killing through CD8^+^ T cell expansion. To the best of our knowledge, one of the first *in vivo* aAPC immunotherapies was performed in mice using silica microspheres bearing either peptide–MHC class I complexes or tumor cell membranes [64]. In mice, these aAPCs could not induce immune responses on their own, but were able to augment responses in the presence of antigen-bearing stimulator tumor cells, which could not be achieved using liposomes as aAPCs [79]. In combination with the chemotherapeutic agent cyclophosphamide, these particles induced regression of established progressing tumors in mice [80]. Variable success was observed for these large multivalent immunogens in Phase I and II clinical trials for the treatment of melanoma and renal cell carcinoma; in some cases, partial responses were induced 81, 82. However, the limited availability of autologous tumor-cell membranes to cover the silica beads restricts the wide applicability of this approach.

In another strategy, microsized polystyrene beads coated with tumor antigen-specific peptide–MHC complexes, anti-CD28 and anti-4-1BB, were injected into tumor-bearing mice. These beads efficiently decreased tumor size and delayed tumor progression 33, 83. Similar results were obtained for magnetic polystyrene microbeads in a mouse tumor model [84]. Interestingly, when compared to spherical PLGA microparticles, ellipsoid particles were most efficient in reducing tumor size and extending survival times in melanoma-bearing mice, again emphasizing the importance of size and shape [67]. Incorporation of IL-2 into biodegradable particles also improved their efficacy and reduced tumor growth kinetics in mice engrafted with B16 tumors [9]. IL-2 encapsulation can also be combined in SLB particles with small-molecule inhibitors, such as TGF-β inhibitors, which increased the activity of intratumoral T cells *in vivo* [85].

Although no *in vivo* toxicities were observed, several of the aAPCs used so far are non-deformable, large, and in some cases non-biodegradable, which may lead to embolisms, making clinical approval for *in vivo* use difficult 9, 16.

Thus, in contrast to microbeads, nanosized particles might be safer and have a better chance of obtaining clinical approval. Particle sizes below 100 nm enable easy entry into the lymphatic system and allow for transportation to the lymph nodes, where particles can gain access to larger numbers of T cells [86]. For example, MHC class II-containing RAFTsomes, which are flexible nanoparticles, were able to induce CD4^+^ T cell responses that prevented EG.7 tumor inoculation and reduced tumor sizes in mice [58]. Unfortunately, generation of these nanoparticles requires DC-derived lipid rafts, and therefore does not completely eliminate the need for isolation and culture of autologous APCs. Alternatively, completely synthetic nanosized aAPCs have been tested for their *in vivo* potential in cancer immunotherapy. Both dextran-coated iron oxide magnetic particles (50–100 nm) and dextran-coated quantum dots (30 nm) can effectively stimulate tumor antigen-specific T cells and inhibit tumor growth *in vivo.* They are also more efficiently distributed and better capable of reaching T cell pools in mice than micro-aAPCs [36].

## Future aAPC development for more potent immunotherapy

The efficacy of signal presentation by aAPCs and the resulting T cell activation are highly dependent on the properties of the materials used. For future aAPC development, the previously discussed properties should be optimally tuned to induce higher clinical responses. *Ex vivo* activation of T cells for subsequent reinfusion into patients has proven most effective with large microsized particles. To further improve aAPCs for *ex vivo* T cell activation, current knowledge about the optimal choice of surface molecules, cytokine release, particle shape, ligand mobility, and ligand orientation should be applied in a microsized system, preferably one that can be quickly separated from the T cell population before reinfusion into a patient. The use of 3D-SLB particles constitutes a promising, yet rarely applied system that would allow for incorporation of these features. For example, future acellular ‘off-the-shelf’ aAPCs could be made by coating ellipsoid PLGA microparticles with GM1-microdomain-containing lipid bilayers, allowing for more optimally shaped and stable particles with ligands pre-clustered in microdomains, high membrane fluidity, and an ability to release paracrine cytokines and small-molecule drugs 15, 85. Furthermore, incorporation of magnetic nanoparticles may be possible for straightforward separation of particles and cells after incubation and expansion. aAPCs that closely mimic features of natural DCs should in this way improve the clinical efficiency of *ex vivo-*generated T cells.

*In vivo*, active aAPC immunotherapy, although more challenging, is also particularly promising. This approach does not depend on autologous T cells, so labor and costs are significantly lower. However, injection of microsized aAPCs may be unsafe, and therefore biocompatible nanoparticulate constructs are preferred. Nanoparticles in the size range 10–100 nm have a favorable biodistribution 36, 86. Similarly, the use of flexible nanoworms is a promising approach for *in vivo* application. These polymers are small in size and have a high degree of flexibility, allowing extensive contacts with the T cell membrane and the dynamic interactions necessary for potent signal transduction [71]. For *in vivo* application of aAPCs, development of nanosized particles combining these properties may be important to boost clinical responses in cancer immunotherapy [15].

## Concluding remarks and future perspectives

The development of aAPCs for cancer immunotherapy is a highly promising approach. The *ex vivo* use of these systems for T cell expansion has significant advantages over the use of autologous APCs, and initial results from clinical studies are encouraging. Therefore, further development of microparticles for this purpose should exploit the effects of size, shape, ligand mobility, and ligand distribution. The development of nanosized aAPCs with improved flexibility, optimal shape, and efficient signal presentation for direct *in vivo* aAPC immunotherapy is desirable and holds marked promise. This approach could eliminate the need for costly and laborious cell culture and lead to broadly accessible ‘off-the-shelf’ cancer immunotherapeutics. This highlights the need for research into nano-aAPCs that exhibit more potent *in vivo* CTL responses.
