# Supplementary material for: Towards efficient cancer immunotherapy: advances in developing artificial antigen-presenting cells
Source: Trends Biotechnol. 2014 Sep;32(9):456–65. doi: 10.1016/j.tibtech.2014.06.007 (PMC4154451; doi:10.1016/j.tibtech.2014.06.007)
Supplement: Supplementary file 1 [file mmc1.docx]

**Supplementary Material**

**Towards efficient cancer immunotherapy: advances in developing artificial antigen-presenting cells**

**Loek J. Eggermont^1^, Leonie E. Paulis^1^, Jurjen Tel^1^, Carl G. Figdor^1^***

1 Department of Tumor Immunology, Radboud University Medical Centre and Radboud Institute for Molecular Life Sciences, Nijmegen, The Netherlands

* Address correspondence and reprint requests to Prof. Dr. Carl G. Figdor, Department of Tumor Immunology, Radboud Institute for Molecular Life Sciences, Radboud University Medical Centre, PO Box 9101, 6500 HB Nijmegen, The Netherlands. Phone: +31 24 3617600. Fax: +31 24 3640339. E-mail address: [c.figdor@ncmls.ru.nl](mailto:c.figdor@ncmls.ru.nl)

**Table S1: Advantages and disadvantages of aAPCs for use in active immunotherapy**

| Type of aAPC | Molecules used | Advantage | Disadvantage | Clinical models | Ref |
| --- | --- | --- | --- | --- | --- |
| Latex microbeads | MHCI, αCD3, CD80, αCD28, CD54, CD83, α4-1BB, 4-1BBL | Easy preparation  Well-defined | Non-biodegadable  Rigid surface |  | [S1–S10] |
|  | MHCI | Antigen-specific | No co-stimulation | H-2K^b^-specific CTL clones C35 and C11  (BALB/c X DBA/2)F1 (H-2d) mouse spleen cells | [S1] |
|  | αCD3, CD80 | Efficient CD4^+^ T cell response | Transient CD8^+^ T cell response  Loss of antigen-specificity  CD8^+^ cell death after 3 days | 6-12-week-old female C57BU6 mice T cells | [S2] |
|  | αCD3, CD80, CD54 | Addition of CD54 increases CD8^+^ T cell responses | No CD8^+^ cell expansion after 3 days  Loss of antigen-specificity | 6-12-week-old female C57BU6 mice T cells | [S5] |
|  | MHCI, αCD28 | Sustained expansion CD8^+^ T cells  No loss of antigen specificity  Can induce antigen-specific CTLs *in vivo* |  | Human CD8+ T lymphocytes  HLA-A2/Kb transgenic mice | [S3]  [S10] |
|  | MHCI, αCD28, α4-1BB | Increased survival memory CD8^+^ T cells  Higher CD8^+^ T cell induction  Increased CTL activity  Anti-tumor activity *in vivo* |  | Human CD8+ T lymphocytes  Naïve and B16 melanoma-bearing B6 mice | [S4]  [S9] |
|  | MHCI, αCD28, CD83 | CD83 may induce additional co-stimulation for CD8^+^ T cells  CD83 may increase survival T cells | Effect of CD83 not compared on beads | Human PBMCs | [S8] |
|  | MHCI, αCD28, CD83, 4-1BBL | CD83 may induce additional co-stimulation for CD8^+^ T cells  CD83 may increase survival | Effect of CD83 not compared on beads | C57BL/6 splenocytes  Naïve and B16 melanoma-bearing B6 mice | [S6] |
| Magnetic microbeads | αCD3, αCD28, MHCI, MHCII, CD80 | Particles have the same immunologic properties as latex microbeads  Easy preparation  Well-defined  Easily removable | Non-biodegadable  Rigid surface |  | [S11–S17] |
|  | MHCII, αCD28 | Induces antigen-specific CD4^+^ T cells |  | Human DR∗0401–Flu HA^+^ CD4^+^ T cells | [S12] |
|  | αCD3, αCD28 | Induces antigen-specific CD4^+^ T cells  T cells generated *in vitro* can induce anticancer response | No CD8^+^ cell expansion *in vitro* after 3 days  Loss of antigen-specificity  Non-specific toxicity T cells *in vivo* | CD4^+^ T cells  Phase I/II adoptive transfer clinical trial, HLA-A2^+^ myeloma patient mononuclear cells  Phase I clinical trial, human donor PBMCs  Phase I clinical trial, donor T cells  Phase I clinical trial, carcinoma patient PBMCs | [S11]  [S13]    [S14]  [S15]  [S16] |
|  | MHCI, CD80 | Induces antigen-specific CD8^+^ T cells  Higher CD8^+^ T cell induction than without co-stimulation  Reduces tumor growth when injected *in vivo* |  | B16 or B16-B7.1 tumor-bearing mice | [S17] |
| Rigid dextran-coated nanoparticles | MHCI, αCD28 | Improved biodistribution  Better *in vivo* activation comparted to microparticles  Safer for *in vivo* use  Induces anti-tumor responses *in vivo* | Rigid surface | 2C and PMel mouse CD8^+^ T cells  Human HLA*0201 PBMCs  B16 melanoma-bearing C57BL/6 male mice | [S18] |
| PLGA microparticles | αCD3, αCD28, MHCI, IL-2 | Easy preparation  Cytokine release  Shape change possible | Rigid surface |  | [S19–S24] |
|  | αCD3, αCD28, IL-2 | Delays tumor growth *in vivo*  IL-2 release leads to higher CD8^+^ T cell induction  Smaller amounts of IL-2 needed  CD8^+^ instead of CD4^+^ induction | Non-specific stimulation | B16 tumor-bearing mice  B6 splenocytes  Mouse C57Bl/6 splenocytes and T cells | [S24]  [S20]  [S21] |
|  | MHCI, αCD28, IL-2 | Antigen-specific T cell stimulation |  | OT-1 CD8^+^ T cells  Human CD8^+^ T cells  pMEL mouse T cells and pMEL mice (inj. with B16 melanoma) | [S20]  [S22]  [S23] |
| Liposomes | MHCII | Easy preparation  Fluid membrane | Unstable  Low T cell activation  No co-stimulation | DO11.10 hybridoma T cells | [S25,S26] |
| RAFTsomes | MHCII, Unknown molecules from RAFT | Fluid membrane  Ligand pre-clustering | Unstable  DC-derived RAFTs  Not well-defined | MF2.2D9 T cell hybridoma cells  OVA-immunized mice CD4^+^ T cells  EG.7 tumor prevention model in C57BL/6 mice | [S27] |
| Microdomain-liposomes | αCD3, αCD28, αLFA-1, MHCII | Fluid membrane  Ligand pre-clustering  Highly controlled | Unstable *in vivo*  Not used for antigen-specificic CD8^+^ T cell activation |  | [S28,S29] |
|  | MHCII, αCD28 | Improved T cell activation compared to non-clustered  liposomes |  | CD4^+^ cells from influenza-immunized donors  human T lymphoid cell line CH7C17 | [S28] |
|  | αCD3, αCD28, αLFA-1 | Improved T cell activation compared to non-clustered  liposomes  Higher T cell induction compared to magnetic beads  αLFA-1 incorporation increases T cell expansion  Preferably expands CD8^+^ T cells with retained antigen-specificity | Non-specific activation | Human donor and melanoma patient T cells | [S29] |
| SLB-particles | MHCI | Stable  Fluid membrane | Not extensively applied | splenocytes from primed CD2F1or C57BL/6 mice | [S30] |
| Large multivalent immunogen (LMI) | Tumor cell membrane proteins | Stable  Fluid membrane | Need for Ag-expressing cell  Undefined surface ligand composition | H-2K^b^-specific CTL clones C35 and C11and (BALB/c X DBA/2)F1 (H-2d) mouse spleen cells  splenocytes from primed CD2F1or C57BL/6 mice  Cloned CTL line C35 and tumor-bearing mice  Mouse peritoneal non-adherent cells and tumor-bearing mice  Tumor-bearing mice  Phase I clinical trial, Stage IV melanoma patients  Phase II clinical trial, Stage IV melanoma patients | [S1]      [S30]  [S31]  [S32]  [S33] [S34]  [S35] |
| Carbon nanotube bundles | αCD3, MHCI, αCD28 | High surface area  Ligand pre-clustering  Negative charge | Very large clusters  Not well-defined |  | [S36–S38] |
|  | αCD3 | Desirable ligand pre-clustering  Tunable surface area | No co-stimulation  Not antigen-specific | B3Z T cells | [S36] |
|  | αCD3, αCD28 | Contains co-stimulation  Desirable ligand pre-clustering  Highly efficient for *ex vivo* T cell induction | Not antigen-specific | Splenocytes from C57BL/6 mouse | [S37] |
|  | MHCI | Desirable ligand pre-clustering  Attachment without MHC denaturing  Enhanced T cell responses | No co-stimulation | Splenocytes from OT-1 mice | [S38] |
| Nanoworms | αCD3 | Semi-flexibility  Nanosized  Good membrane contact  Applicable *in vivo*  More potent than rigid particles | Not antigen-specific  No co-stimulation | Human T cells | [S39] |

**Supplementary references:**

S1 Mescher, M.F. (1992) Surface contact requirements for activation of cytotoxic T lymphocytes. *J. Immunol.* 149, 2402–2405

S2 Deeths, M.J. and Mescher, M.F. (1997) B7-1-dependent co-stimulation results in qualitatively and quantitatively different responses by CD4+ and CD8+ T cells. *Eur. J. Immunol.* 27, 598–608

S3 Oelke, M. *et al.* (2003) Ex vivo induction and expansion of antigen-specific cytotoxic T cells by HLA-Ig-coated artificial antigen-presenting cells. *Nat. Med.* 9, 619–24

S4 Rudolf, D. *et al.* (2008) Potent costimulation of human CD8 T cells by anti-4-1BB and anti-CD28 on synthetic artificial antigen presenting cells. *Cancer Immunol. Immunother.* 57, 175–83

S5 Deeths, M.J. and Mescher, M.F. (1999) ICAM-1 and B7-1 provide similar but distinct costimulation for CD8 + T cells , while CD4 + T cells are poorly costimulated by ICAM-1. *Eur. J. Immunol.* 29, 45–53

S6 Lu, X. *et al.* (2008) Adoptive transfer of pTRP2-specific CTLs expanding by bead-based artificial antigen-presenting cells mediates anti-melanoma response. *Cancer Lett.* 271, 129–139

S7 Oelke, M. and Schneck, J.P. (2010) Overview of a HLA-Ig based “‘ Lego-like system ’” for T cell monitoring , modulation and expansion. *Immunol. Res.* 47, 248–256

S8 Jiang, X. *et al.* (2007) HLA Tetramer Based Artificial Antigen-Presenting Cells Efficiently Stimulate CTLs Specific for Malignant Glioma. *Clin. Cancer Res.* 13, 7329–34

S9 Shen, C. *et al.* (2013) Latex bead-based artificial antigen-presenting cells induce tumor-specific CTL responses in the native T-cell repertoires and inhibit tumor growth. *Immunol. Lett.* 150, 1–11

S10 Shen, C. *et al.* (2007) Induction of tumor antigen-specific cytotoxic T cell responses in naïve mice by latex microspheres-based artificial antigen-presenting cell constructs. *Cell. Immunol.* 247, 28–35

S11 Levine, B.L. *et al.* (1997) Effects of CD28 costimulation on long-term proliferation of CD4+ T cells in the absence of exogenous feeder cells. *J. Immunol.* 159, 5921–30

S12 Maus, M. V *et al.* (2003) HLA tetramer-based artificial antigen-presenting cells for stimulation of CD4+ T cells. *Clin. Immunol.* 106, 16–22

S13 Rapoport, A.P. *et al.* (2009) Rapid immune recovery and graft-versus-host disease-like engraftment syndrome following adoptive transfer of Costimulated autologous T cells. *Clin. Cancer Res.* 15, 4499–507

S14 Hardy, N.M. *et al.* (2011) Phase I trial of adoptive cell transfer with mixed-profile type-I/type-II allogeneic T cells for metastatic breast cancer. *Clin. Cancer Res.* 17, 6878–87

S15 Porter, D.L. *et al.* (2006) A phase 1 trial of donor lymphocyte infusions expanded and activated ex vivo via CD3/CD28 costimulation. *Blood* 107, 1325–31

S16 Lum LG, LeFever AV, Treisman JS, Garlie NK, H.J.J. (2001) Immune Modulation in Cancer Patients After Adoptive Transfer of Anti-CD3/Anti-CD28-Costimulated T Cells-Phase I Clinical Trial. *J. Immunother.* 24, 408–419

S17 Ugel, S. *et al.* (2009) In vivo administration of artificial antigen-presenting cells activates low-avidity T cells for treatment of cancer. *Cancer Res.* 69, 9376–84

S18 Perica, K. *et al.* (2014) Nanoscale Artificial Antigen Presenting Cells for T Cell Immunotherapy. *Nanomedicine* 10, 119–129

S19 Fahmy, T.M. *et al.* (2005) Surface modification of biodegradable polyesters with fatty acid conjugates for improved drug targeting. *Biomaterials* 26, 5727–5736

S20 Steenblock, E.R. and Fahmy, T.M. (2008) A comprehensive platform for ex vivo T-cell expansion based on biodegradable polymeric artificial antigen-presenting cells. *Mol. Ther.* 16, 765–772

S21 Steenblock, E.R. *et al.* (2011) An artificial antigen-presenting cell with paracrine delivery of IL-2 impacts the magnitude and direction of the T cell response. *J. Biol. Chem.* 286, 34883–34892

S22 Han, H. *et al.* (2011) A novel system of artificial antigen-presenting cells efficiently stimulates Flu peptide-specific cytotoxic T cells in vitro. *Biochem. Biophys. Res. Commun.* 411, 530–5

S23 Sunshine, J.C. *et al.* (2014) Particle shape dependence of CD8+ T cell activation by artificial antigen presenting cells. *Biomaterials* 35, 269–277

S24 Steenblock, E.R. *et al.* (2009) Antigen presentation on artificial acellular substrates: modular systems for flexible, adaptable immunotherapy. *Expert Opin. Biol. Ther.* 9, 451–464

S25 Prakken, B. *et al.* (2000) Artificial antigen-presenting cells as a tool to exploit the immune “synapse”. *Nat. Med.* 6, 1406–10

S26 Mallet-Designe, V.I. *et al.* (2003) Detection of low-avidity CD4+ T cells using recombinant artificial APC: following the antiovalbumin immune response. *J. Immunol.* 170, 123–31

S27 Ding, Q. *et al.* (2013) RAFTsomes containing epitope-MHC-II complexes mediated CD4+ T cell activation and antigen-specific immune responses. *Pharm. Res.* 30, 60–69

S28 Giannoni, F. *et al.* (2005) Clustering of T Cell Ligands on Artificial APC Membranes Translocation to the T Cell Plasma Membrane. *J. Immunol.* 174, 3204–3211

S29 Zappasodi, R. *et al.* (2008) The effect of artificial antigen-presenting cells with preclustered anti-CD28/-CD3/-LFA-1 monoclonal antibodies on the induction of ex vivo expansion of functional human antitumor T cells. *Haematologica* 93, 1523–1534

S30 Goldstein, S. a and Mescher, M.F. (1986) Cell-sized, supported artificial membranes (pseudocytes): response of precursor cytotoxic T lymphocytes to class I MHC proteins. *J. Immunol.* 137, 3383–3392

S31 Mescher, M.F. and Savelieva, E. (1997) Stimulation of Tumor-Specific Immunity Using Tumor Cell Plasma Membrane Antigen. *Methods* 12, 155–164

S32 Rogers, J. and Mescher, M.F. (1992) Augmentation of in vivo cytotoxic T lymphocyte activity and reduction of tumor growth by large multivalent immunogen. *J. Immunol.* 149, 269–76

S33 Mescher, M.F. and Rogers, J.D. (1996) Immunotherapy of established murine tumors with large multivalent immunogen and cyclophosphamide. *J. Immunother. Emphasis Tumor Immunol.* 19, 102–12

S34 Mitchell, M.S. (2004) Phase I Trial of Large Multivalent Immunogen Derived from Melanoma Lysates in Patients with Disseminated Melanoma. *Clin. Cancer Res.* 10, 76–83

S35 Dudek, A.Z. *et al.* (2008) Autologous large multivalent immunogen vaccine in patients with metastatic melanoma and renal cell carcinoma. *Am. J. Clin. Oncol.* 31, 173–81

S36 Fadel, T.R. *et al.* (2008) Enhanced cellular activation with single walled carbon nanotube bundles presenting antibody stimuli. *Nano Lett.* 8, 2070–6

S37 Fadel, T.R. *et al.* (2010) Clustering of stimuli on single-walled carbon nanotube bundles enhances cellular activation. *Langmuir* 26, 5645–54

S38 Fadel, T.R. *et al.* (2013) Adsorption of multimeric T cell antigens on carbon nanotubes: effect on protein structure and antigen-specific T cell stimulation. *Small* 9, 666–72

S39 Mandal, S. *et al.* (2013) Therapeutic nanoworms: towards novel synthetic dendritic cells for immunotherapy. *Chem. Sci.* 4, 4168
